# Cryptic Genetic Variation for *Arabidopsis thaliana* Seed Germination Speed in a Novel Salt Stress Environment

**DOI:** 10.1534/g3.116.033944

**Published:** 2016-08-18

**Authors:** Wei Yuan, Jonathan M. Flowers, Dustin J. Sahraie, Michael D. Purugganan

**Affiliations:** * Department of Biology, Center for Genomics and Systems Biology, New York University, New York 10003; † Center for Genomics and Systems Biology, New York University Abu Dhabi Research Institute, New York University Abu Dhabi, United Arab Emirates

**Keywords:** salinity tolerance, salt stress, QTL, bulk segregant analysis, abiotic stress

## Abstract

The expansion of species ranges frequently necessitates responses to novel environments. In plants, the ability of seeds to disperse to marginal areas relies in part to its ability to germinate under stressful conditions. Here we examine the genetic architecture of *Arabidopsis thaliana* germination speed under a novel, saline environment, using an Extreme QTL (X-QTL) mapping platform we previously developed. We find that early germination in normal and salt conditions both rely on a QTL on the distal arm of chromosome 4, but we also find unique QTL on chromosomes 1, 2, 4, and 5 that are specific to salt stress environments. Moreover, different QTLs are responsible for early *vs.* late germination, suggesting a temporal component to the expression of life history under these stress conditions. Our results indicate that cryptic genetic variation exists for responses to a novel abiotic stress, which may suggest a role of such variation in adaptation to new climactic conditions or growth environments.

Germination is a key life history stage in plants. In the model species *Arabidopsis thaliana*, germination has been shown to determine key aspects of organismal fitness (Donohue 2002; Donohue *et al.* 2010, 2005b). Seasonal cueing of germination, for example, has been shown to have major consequences both for shaping life history strategies in this species and for local adaptation (Donohue 2002; Donohue *et al.* 2005a). One key germination trait is germination speed (Yuan *et al.* 2016), *i.e.*, the time lapse for a nondormant seed to germinate upon the reception of cue, or the time lapse for an isogenic seed lot to reach 50% germination. Though little is known about germination speed in *A. thaliana*, comparative studies with various other species correlates this trait with fitness under fluctuating environments (Parsons 2012), competitiveness (Miller *et al.* 1994; Weinig 2000; Dubois and Cheptou. 2012), and establishment during range expansion (Graebner *et al.* 2012; Beckman *et al.* 2011). In a previous study, we demonstrated that germination speed is variable among *A. thaliana* accessions, and identified QTL for this trait using an Extreme QTL (X-QTL) mapping strategy (Yuan *et al.* 2016).

As sessile organisms, plants rely on seed dispersal to alleviate resource competition and seed predation in local environments. Seed dispersal also enables migration to novel resource patches. Given the ability of plants to disperse their seeds, they can presumably encounter novel environments to which they have had no previous adaptive history. Should a seed manage to germinate in these novel environments, the habitat can determine the strength and types of selection the plant must experience throughout all stages of its life cycle.

Response to novel environments underlies the process of local adaptation, range expansion, and ecological speciation (Atwell *et al.* 2014). Recent studies have focused on characterizing the types of traits that serve as the primary targets of selection under novel environments (Atwell *et al.* 2014; Dutilleul *et al.* 2014; Handelsman *et al.* 2013). Life history traits and the genotype-by-environment (G×E) interactions in the expression of life history stages are believed to be crucial in determining the likely path of adaptation and range expansion (Handelsman *et al.* 2013; Seiter and Kingsolver 2013; Cameron *et al.* 2013). However, relatively little is known regarding the genetics of life history traits in response to novel environments, including the types of genes that are likely under selection. Understanding the genetics of novel environmental response is important in examining how selection acts when organisms disperse to new environments at the edges of species ranges, or experience new climactic regimes.

The ability to respond to novel environment differs within natural populations, depending on the amount of standing genetic variation for the trait under selection (Volis *et al.* 2016; Etterson and Shaw 2001). Standing genetic variation can underlie phenotypic diversity, but recently it has been shown that cryptic genetic variation can be important under certain environments or in genetic backgrounds (Paaby and Rockman 2014; McGuigan *et al.* 2011; Jarosz and Lindquist 2010; Hayden *et al.* 2011). The lack of selection on cryptic genetic variation in native environments allows them to accumulate within populations (Paaby and Rockman 2014), and enable them to respond rapidly to selective pressures under novel environments.

Salinity is an abiotic soil condition caused by natural processes such as mineral weathering, past or present proximity to coastal waters, or through anthropogenic processes such as irrigation (Rhoades and Loveday 1990). Worldwide, saline soils are found over ∼323 million hectares, or ∼7% of global land area (Lynch and Clair 2004). Soil salinity is rapidly becoming a major agricultural issue, but for wild species it also represents a marginal soil environment, which can exert strong adaptive pressure (Assman 2013; Lefebvre *et al.* 2009). The model wild species *A. thaliana*, for example, is not known to have widespread adaptation to saline environment, though as a colonizing species, there is a possibility for an *A. thaliana* individual and/or population to encounter a saline environment. Moreover, local adaptation in coastal regions was shown to be possible (Rus *et al.* 2005; Baxter *et al.* 2010). Salinity thus represents a novel but relevant environment for most *Arabidopsis* populations.

Relatively little is known about salinity tolerance during the germination stage in *A. thaliana*, although evidence suggests that the genetic mechanism of salt tolerance during germination is quite different from the vegetative growth stage (Quesada *et al.* 2002; Lefebvre *et al.* 2009). Several studies have identified different genomic regions responsible for salt tolerance in both germination and vegetative growth stages (for example, Clerkx *et al.* 2004; DeRose-Wilson and Gaut 2011; Joosen *et al.* 2010; Vallejo *et al.* 2010; Guo *et al.* 2016; Galpaz and Reymond 2010; Quesada *et al.* 2002; Rus *et al.* 2005; Baxter *et al.* 2010). Moreover, *RAS1*, a large-effect negative regulator of germination salt tolerance, was cloned in *A. thaliana* (Ren *et al.* 2010). This gene is distinct from the *AtHKT1* locus, which is a salinity tolerance gene during vegetative growth (Rus *et al.* 2005).

In this study, we examine cryptic genetic variation for germination speed under novel high-salt environments by mapping germination speed QTL in *A. thaliana* using the X-QTL approach (Ehrenreich *et al.* 2010; Yuan *et al.* 2016). X-QTL mapping applies bulk segregant analysis and high-throughput genotyping to very large mapping populations (∼10^5^–10^6^ individuals), and can provide greater power in detecting moderate- to small-effect QTL (Ehrenreich *et al.* 2010; Yuan *et al.* 2016). By identifying germination speed QTL under saline environments and comparing them to loci found in normal growth conditions (Yuan *et al.* 2016), we can begin to dissect the genetic architecture of *A. thaliana* in response to a novel and ecologically important environment. We can then catalog the extent to which cryptic genetic variation contributes to potential life history adaptation to a stressful environment, and uncover these variants through induced artificial selection.

## Materials and Methods

### Plant materials

The mapping population was generated using Bs-2 (CS6628) and Col-0 (CS6673) accessions of *A. thaliana* as parental accessions. For plant cultural conditions, seed storage condition, and a detailed description of the mapping population see Yuan *et al.* (2016).

### Phenotyping the germination stages

Germination *sensu stricto* is defined as the moment when the radicle (embryonic root) ruptures the endosperm and protrudes through the seed coat. However, for the convenience of our study, we used a phase early in seedling growth, cotyledon expansion, as our marker for germination. All our ensuing usage of the word “germination” therefore refers to cotyledon expansion. For each parental accession, 50 mg (∼2000) dry seeds were evenly spread onto an 80-cm^2^ gridded square petri dish (Fisher brand) containing Murashige–Skoog basal solid media with or without 250 mM NaCl supplement. A sterilized 500-μm nylon mesh was applied to the interface of the medium and the seeds to prevent seed clumping. Three square grid units (1.74 cm^2^ each) were randomly chosen from each plate, and the germination stage of individual seeds within the chosen grid units were closely examined under a stereoscopic microscope (Stereozoom3; Bausch & Lomb) using 10× magnification at 6-hr time intervals. The number of seeds reaching the testa rupture, radicle protrusion, cotyledon greening, and cotyledon expansion phases at each time point were recorded.

### Phenotyping germination speed

Triplicate scoring was carried out for each accession under both no-salt and salt (250 mM NaCl) conditions, and each replicate contained 50 mg (∼2000) dry seeds. Pretreatment, plating, and stratification followed Yuan *et al.* (2016). Germination was scored by visually identifying, counting, and removing germinants that reached the cotyledon expansion phase. For the no-salt condition the scoring lasted for 5 d at 6-hr intervals, and for saline condition, daily for 15 consecutive days.

### Selection experiment and mapping

Selection for salt-tolerant germinants was carried out in duplicate of ∼100,000 F_3_ populations. Seeds were harvested, after-ripen, sterilized, plated, stratified, and placed into the chamber conditions as described in Yuan *et al.* (2016). Germinants reaching the cotyledon expansion phase were visually identified, counted, and collected from the plate daily. The sampling process lasted 15 d for each replicate, until very few (*n* < 100) individuals germinated each day. Approximately 23% of F_3_ seeds (23,000 individuals) had germinated by the end of the experiment.

The germinants were grouped into early [0–7 d after stratification (DAS); ∼0-5th percentile of total seed], medium (8–12 DAS; ∼5th–20th percentile of total seed), and late (13–15 DAS; ∼20th–23rd percentile of total seed) cohorts, each corresponding to the lagging, linear, and plateau phase of the sigmoidal cumulative germination curve. DNA was extracted from each of the pooled cohorts, and a fourth pool consisting of total DNA of all ∼23,000 F_3_ salt-tolerant germinants was made by proportionally mixing together three DNA pools according to their DNA concentration and the number of individuals in each pool.

We mapped for salt-tolerance germination QTL using the X-QTL method (Ehrenreich *et al.* 2010) as developed for *A. thaliana* (Yuan *et al.* 2016). We determined allele frequency for 30,389 single nucleotide polymorphisms (SNPs) using a custom-designed isothermal Agilent microarray DNA chip we previously developed for *A. thaliana* (Yuan *et al.* 2016). Dye-swap hybridization was carried out for each of the four pools, with pool-extracted DNA from ∼2000 random F_2_ recombinants (equal in size to the founder population, and at the same germination stage) as reference. Random F_2_ individuals were used as reference to control for the possible segregation distortion in the founder population that might have biased our allele frequency estimates at specific SNP markers.

The microarray-based allele frequency estimate of each salt-tolerant germinant DNA pool was compared against that of a control DNA pool of ∼100,000 F_3_ germinants, sampled under no-salt conditions, using a sliding window, three-way nested ANOVA model described previously (Yuan *et al.* 2016). Statistical significance for QTL peaks was established via permutation. We then identified SNPs above the significance threshold, and defined QTL as regions with consecutive SNPs that exceed the threshold. In cases (*e.g.*, peak cluster on chromosome 4) where two or more groups of consecutively significant SNPs were <1 Mbp apart, the entire region was considered as one large QTL.

### Germination curve fitting

Raw cumulative germination fraction of the parental accessions and the F_3_ mapping populations at each time point were used to fit a four-parameter Hill function (4PHF) models (El-Kassaby *et al.* 2008; Joosen *et al.* 2010). The parameters for each independent experiment were solved using the nls() function in R (R Core Team 2014) package chemCal (Ranke 2014), and with the least sum-of-squares method. Germination speed for each accession was calculated by averaging the final estimates of time to reach 50% germination from all three replicates.

### Data availability

Bulk-segregant microarray genotyping data of early, medium and late salt-tolerant germination cohorts, and the entire salt germinant pool are available in Supplemental Material, File S1.

## Results

### Salinity increases the difference in germination speed between parental accessions

An F_3_ mapping population was created by crossing two natural accessions: Col-0 (CS6673) and Bs-2 (CS6628). Neither of the parental accessions originated from a coastal area or a known inland saline area; Bs-2 being from Basel, Switzerland and Col-0 being genetically closest to populations from Gückingen, Germany (Nordborg *et al.* 2005). Therefore it is unlikely that either accession came from a population that had prior adaptation history to salinity, and salinity likely represents a novel abiotic environment for these two accessions. Despite this, Quesada *et al.* (2002) reported significant difference in germination salt tolerance between the accessions, with Bs-2 being tolerant and Col-0 being sensitive to salt (250 mM NaCl) treatment on soil at the germination stage.

**Figure 1:**
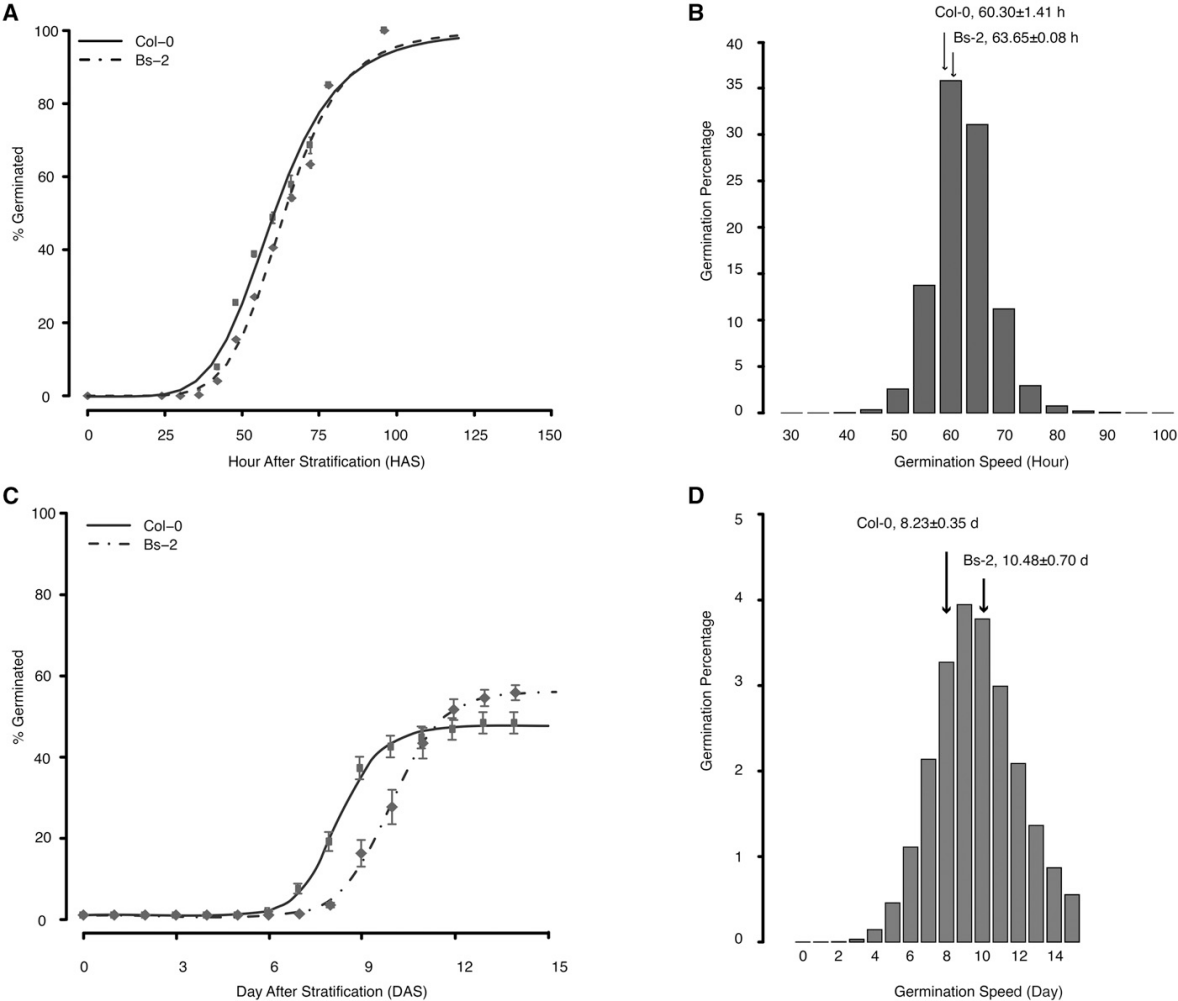
Germination speed of the founder accessions and their F_3_ populations under no-salt condition and extreme salinity. (A) Cumulative germination percentage of the founder accessions (Bs-2 and Col-0). Gray dots show the percentage germinated at each sampling time point, with the center of the dot indicating mean germination percentage, and the error bar showing the SE. Where error bars cannot be seen, they are because the size of the error bars at those time points are smaller than the size of the dots. The black lines show the 4PHF fitting result. Raw data were fitted to 4PHF with 100 maximum iteration and least sum-of-square method. Data from Yuan *et al.* (2016). (B) Histogram showing the distribution of germination speed in ∼100,000 Bs-2 × Col-0 F_3_ population. Data for plotting the histogram was derived from fitting raw F_3_ germination data to 4PHF. The germination speed of Bs-2 and Col-0 were indicated by the arrows. The F_3_ population exhibits transgressive segregation in germination speed Data from Yuan *et al.* (2016). (C) Cumulative germination percentage of the founder accessions under extreme salinity (250 mM NaCl). Compared to germination under no-salt condition (Figure 1A), both accessions showed significant delay in onset of germination (*P* < 2.2 × 10^−16^), reduction in final germination percentage (*P* = 5.3 × 10^−10^), and a greater difference between their germination speed (*P* = 2.7 × 10^−9^). (D) Histogram showing the distribution of germination speed in ∼100,000 F_3_ population under extreme salinity. The higher tail was cut off at the end of the experiment. The transgressive segregation of germination speed under salt is evident in the F_3_ population.

We measured the germination speed of these two accessions under no-salt (Murashige–Skoog basal solid media) and extremely saline (Murashige–Skoog basal solid media supplemented with 250 mM NaCl) conditions. The latter is equivalent to 42% of seawater, and would be considered a highly stressful abiotic environment. As reported in our previous publication, the accessions exhibited significant yet small differences in their germination speed under no-salt conditions (difference: 3.35 hr, *P* < 0.05; Figure 1A, data from Yuan *et al.* 2016).

The addition of salt dramatically delayed the onset of germination of both parental accessions from ∼30 hr after stratification to ∼5–6 DAS, and drastically reduced the final germination percentage to ∼40–50%. More importantly, the saline environment amplified the difference in germination speeds between the accessions, from ∼3 hr to 2.25 d (Figure 1C). Both genotype effect and G×E effect are significant (*P*_genotype_ = 2.71 × 10^−9^; *P*_G×E_ = 1.95 × 10^−9^, two-way ANOVA) in explaining the difference in germination speed observed between parents under different treatments.

To examine the germination behavior in the F_3_ population, ∼100,000 F_3_ seeds were evenly spread onto the surface of Murashige–Skoog basal solid media supplemented with 250 mM NaCl at a density of ∼40 seeds/cm^2^. Germinants reaching the cotyledon expansion phase were counted daily and removed from the plate. The germination speed of the F_3_ population exhibited transgressive segregation (Figure 1D), with sporadic germinants observed as early as 3 DAS. Approximately 23,000 germinants (23%) were collected in a 15-d period, and scoring of germination stopped at 15 d as germination reached a plateau phase.

### The increase in germination speed variation is via differential timing of each developmental stage

For the convenience of our study, we use a phase early in seedling growth, cotyledon expansion, as our marker for germination. According to our expansive definition, the germination process thus consists of several physiological/developmental stages: testa rupture, radicle protrusion, cotyledon greening, and cotyledon expansion.

**Figure 2:**
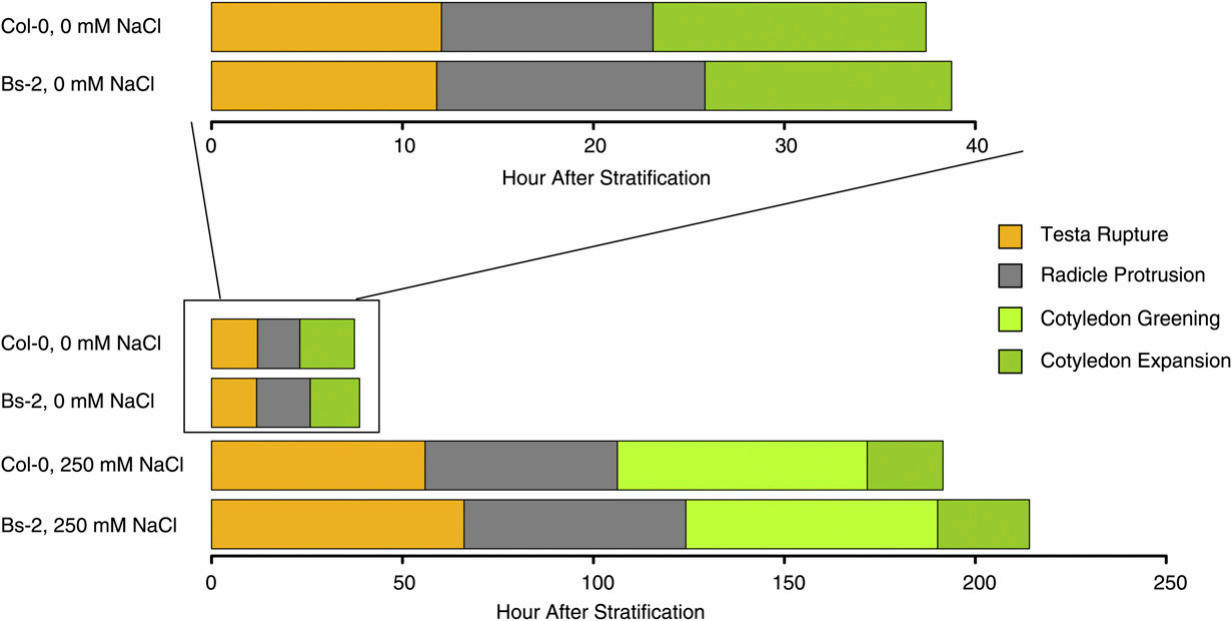
Schedule and duration of four stages during germination in the founder populations. The mean time for Bs-2 and Col-0 seeds sown onto no-salt or salt (250 mM NaCl) media plates to reach testa rupture (gold), radicle protrusion (gray), cotyledon greening (light green), and cotyledon expansion (dark green) are shown. The cotyledon greening phase was too transient to record with our time interval under no-salt conditions. The top panel shows a magnified view of the no-salt condition, under which only the schedules of radicle protrusion and cotyledon expansion were significantly different between the accessions (*P* < 0.005), while schedules of all four stages were significantly different between the accessions under saline condition (*P* < 0.05).

The timing for each of the four stages during the germination process for the parental accessions is measured in both no-salt and saline conditions (Figure 2). In no-salt conditions, both accessions reached 100% germination at the end of the experiment, and the schedule of testa rupture was the same between Bs-2 and Col-0 (Student’s *t*-test, *P* > 0.62; see Table 1). The Bs-2 accession, however, showed later progression into radicle protrusion and cotyledon expansion stages (Student’s *t*-test, *P* < 0.005). The delay in radicle protrusion and cotyledon expansion in Bs-2 likely explains the overall slower germination for this accession compared to Col-0.

**Table 1:** Cumulative time (in hours) for the founder populations to reach four distinct phases during germination under no-salt and saline conditions

Condition	Stage	Accession	Average/hr	SD	*P* Value
0 mM NaCl (no-salt)	Testa rupture	Bs-2	11.789	0.227	0.6211
		Col-0	12.043	0.79	
	Radicle protrusion	Bs-2	25.836	0.5	0.0011**
		Col-0	23.116	0.25	
	Cotyledon expansion	Bs-2	38.761	0.348	0.0036**
		Col-0	37.419	0.152	
250 mM NaCl (salt)	Testa rupture	Bs-2	66.133	1.62	0.0004***
		Col-0	55.907	0.235	
	Radicle protrusion	Bs-2	124.202	1.687	0.0085*
		Col-0	106.282	6.209	
	Cotyledon greening	Bs-2	190.125	1.957	0.0177*
		Col-0	171.707	8.846	
	Cotyledon expansion	Bs-2	214.168	0.59	0.0016**
		Col-0	191.542	5.125	

* *P* < 0.05; ** *P* < 0.005; *** *P* < 0.0005.

The salinity-induced difference in germination speed between accessions is likely the combined effects of timing differences through all the developmental stages. In saline conditions, the difference in timing of every single stage of development was amplified between the two parental accessions, with the Bs-2 accession consistently exhibiting slower progress through the germination stages (Student’s *t*-test, *P* < 0.05 for all stages; Table 1). Notably, under extreme salt stress, the germination of many plants in both accessions was arrested at the cotyledon greening phase without ever progressing into cotyledon expansion. The ratio of individuals at cotyledon greening phase *vs.* cotyledon expansion phase approached a constant value for Col-0 at 6 d (∼40% greening *vs.* 45% expansion), and for Bs-2 at 9 d (∼30% greening *vs.* 60% expansion). Also, the slower germinating Bs-2 accession exhibited acceleration in cotyledon greening after 6 d, exceeding Col-0 in the fraction of seeds that reached this germination stage, and later on exhibited a greater percentage of plants reaching cotyledon expansion.

### Germination speed QTL under salinity stress

**Figure 3:**
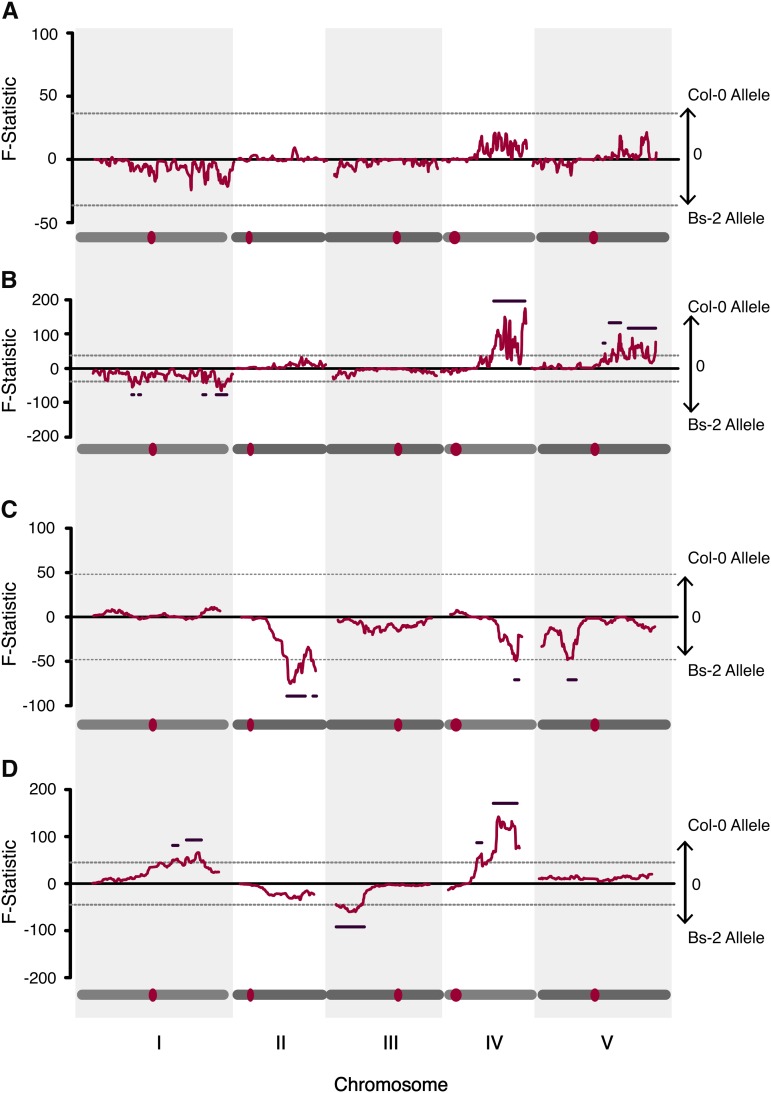
Genetic architecture of germination speed under extreme salinity in *Arabidopsis thaliana*. Allele frequency of (A) all germinants, (B) early germinants, (C) late salt germinants, and (D) early germinants under no-salt condition. Figure 3D is generated with data from Yuan *et al.* (2016). Data were fitted to a sliding window, three-way nested ANOVA model. The F-statistic was plotted along the genome, with positive values indicating bias for the Col-0 allele and negative values for the Bs-2. Significance threshold (shown as gray dashed lines) and optimal window size were established via permutation. The dark thin bars within each plot indicate the QTL regions, and the gray bars underneath each panel indicate the chromosomes, and the red dots indicate the position of the centromere.

In a previous study, we developed an X-QTL mapping platform for *A. thaliana* and used this to identify QTL for germination speed under no-salt conditions. To compare germination speed QTL between no-salt and salt stress conditions, we collected the early germinants (first 5% of F_3_ individuals) under salt conditions in our experiment. The DNA of this early germinant pool was extracted and hybridized to a custom isothermal SNP microarray (Yuan *et al.* 2016) to quantify allele frequency changes that are associated with QTL for germination speed under saline conditions (Figure 3B).

Using this X-QTL method, we were able to identify one peak on chromosomes 4 and three on chromosome 5, all with allele frequency biased toward the Col-0 allele for early germination. Four peaks were also identified on chromosome 1, biased toward the Bs-2 allele. In addition, multiple regions on chromosomes 2 and 3 were identified that just reached the significance threshold. The position and size of the QTL are shown in Table 2.

**Table 2:** Positions of germination speed under salt X-QTL, and their overlap with previously identified salt germination QTL

QTL	Chromosome	Range/Mbp	Size/Mbp	Overlap	Trait
SaltEarlyQ1.1	1	8.58–10.78	2.2	Clerkx *et al.* 2004	Ler/Sha RIL, 150 mM NaCl, % germination
				DeRose-Wilson and Gaut 2011	Ler/Cvi RIL, % germination, 150 mM
				DeRose-Wilson and Gaut 2011	Ler0/Col4 RIL, % cotyledon expansion
				Joosen *et al.* 2010	Bay0/Sha RIL, 100 mM NaCl, Gmax
				Joosen *et al.* 2010	Bay0/Sha RIL, 100 mM NaCl, AUC
				Vallejo *et al.* 2010	Bay0/Sha RIL, 50 mM NaCl, % germination at 4 d
				Guo *et al.* 2016	Tsu1/Sha F2, 150 mM NaCl, cotyledon greening
				Galpaz and Reymond 2010	Sha/Ler RIL, 175 mM NaCl, % germinated at 10 d after sowing
SaltEarlyQ1.2	1	11.92–12.20	0.18	Quesada *et al.* 2002	Ler0/Col4 RIL, 250 mM NaCl, % germination at 15 d
				Guo *et al.* 2016	Tsu1/Sha F2, 150 mM NaCl, cotyledon greening
SaltEarlyQ1.3	1	23.60–24.59	0.99	Guo *et al.* 2016	Tsu1/Sha F2, 150 mM NaCl, cotyledon greening
SaltEarlyQ1.4	1	26.29–28.75	2.46		
SaltEarlyQ4.1	4	10.87–18.08	7.21	Quesada *et al.* 2002	Ler0/Col4 RIL, 250 mM NaCl, % germination at 15 d
				DeRose-Wilson and Gaut 2011	Ler/Cvi RIL, % germination, 150 mM
				DeRose-Wilson and Gaut 2011	Ler0/Cvi RIL, % cotyledon expansion
SaltEarlyQ5.1	5	15.88–16.09	0.21	Joosen *et al.* 2010	Bay0/Sha RIL, 100 mM NaCl, AUC
SaltEarlyQ5.2	5	17.33–25.18	7.85	Clerkx *et al.* 2004	Ler/Sha RIL, 150 mM NaCl, % germination
				DeRose-Wilson and Gaut 2011	Ler/Cvi RIL, % germination, 150 mM
				DeRose-Wilson and Gaut 2011	Ler0/Col4 RIL, T50
				Joosen *et al.* 2010	Bay0/Sha RIL, 100 mM NaCl, Gmax
				Joosen *et al.* 2010	Bay0/Sha RIL, 100 mM NaCl, T50
				Joosen *et al.* 2010	Bay0/Sha RIL, 100 mM NaCl, AUC
				Vallejo *et al.* 2010	Bay0/Sha RIL, 50 mM NaCl germination at 4 d
				Galpaz and Reymond 2010	Sha/Col RIL, 175 mM NaCl, % germinated at 10 d after sowing
SaltEarlyQ5.3	5	26.34–26.66	0.32		
SaltLateQ2.1	2	10.76–15.01	4.25	Guo *et al.* 2016	Tsu1/Sha F2, 150 mM NaCl, cotyledon greening
SaltLateQ2.2	2	16.32–17.47	1.15		
SaltLateQ4.1	4	14.89–15.29	0.4	DeRose-Wilson and Gaut 2011	Ler0/Cvi RIL, % cotyledon expansion
SaltLateQ5.1	5	6.90–6.98	0.08	Joosen *et al.* 2010	Bay0/Sha RIL, 100 mM NaCl, T50
				Joosen *et al.* 2010	Bay0/Sha RIL, 100 mM NaCl, AUC
				Guo *et al.* 2016	Tsu1/Sha F2, 150 mM NaCl, cotyledon greening

Three rows are empty for Overlap and Trait because no overlap with previous publications were identified for those QTLs. Gmax, maximum germination percentage; AUC, area under cumulative germination curve.

We compared the X-QTL mapping results of the early cohorts (the initial 5000 germinants) in the saline conditions *vs.* no-salt conditions (Figure 3, B and D). While only three peak clusters on chromosomes 1, 3, and 4 were significant in no-salt conditions, multiple peaks on chromosomes 1, 4, and 5 were identified for rapid germination under salt. Among all peak regions that were significant, only the peaks on the right arm of chromosome 4 were shared between the rapid germinating cohorts under both conditions, and the Col-0 allele was favored in both cases.

### Time-cohort germination QTL under salinity stress

Unlike under no-salt conditions, germination under salinity stress occurred over a prolonged time period that spanned >12 d. Given this prolonged period, we could examine what QTL were associated with germination under different time periods during salinity stress.

We identified two germination cohorts for analysis. The first 5% that germinated of the entire F_3_ population (see above) were designated the early cohort, and corresponds to the lagging phase of the cumulative germination curve. The late cohort corresponds to the plateau phase of germination curve, and constitutes the 20th–23rd percentiles that germinated of the same F_3_ populations.

Three genomic regions exhibited a response to selection in the late cohort, on chromosome 2, 4, and 5 respectively, and all three regions favor the Bs-2 allele (Figure 3C). In this late cohort, the peak on chromosome 2 passed the permutation threshold, while the ones on chromosome 4 and 5 narrowly reached significance. Interestingly, overlapping regions on chromosome 2 and 4 also showed response to selection in the early cohort, although the allele frequency bias on chromosome 2 is not significant; however the allele favored for rapid germination in the early cohort was Col-0.

Finally, we examined whether there was a genetic basis for the ability to germinate under salt stress (as opposed to germination speed). We pooled DNA isolated from all time cohorts, representing the ∼23% of F_3_ recombinants that germinated under salt across the 15 d of the experiment. In this population, we observed allele frequency skews on chromosomes 1, 4, and 5. However, none of these were significant as determined by permutation (Figure 3A). This could be attributable to the different QTL associated with germination at different time points (*e.g.*, early *vs.* late cohorts), some of which are attributed to QTL alleles with different directions in phenotypic effect.

## Discussion

Salinity is a novel environment for the majority of *A. thaliana* populations, and previous studies have examined natural variation for salt tolerance in this species in both germination and vegetative growth stages. Salt tolerance during vegetative growth, for example, has been shown to be under control of few loci of large effect (*e.g.*, Rus *et al.* 2005; Quesada *et al.* 2002; Katori *et al.* 2010). The *RAS1* locus was cloned in a Ler × Sha recombinant inbred line (RIL) mapping population, and a naturally occurring premature stop codon in the Sha allele leads to decreased salt tolerance (Ren *et al.* 2010). Also, a weak allele of the sodium transporter *AtHKT1* gene shows altered expression patterns in two coastal populations of *A. thaliana* that confers salt tolerance (>30% variation explained) during vegetative growth. A broader survey of >300 natural populations shows an adaptive cline of salt tolerance that was driven by the distribution of the weak *AtHKT1* allele.

Germination of dispersed seeds is crucial for the range expansion and establishment of plants in novel environments, and germination speed affects the fitness of plants under fluctuating environments. Much less is known about the genetic basis of salt tolerance during germination. We identified nine QTL studies (see Table 2 for the full list) that examined the genetic architecture of *A. thaliana* germination salt tolerance. The number of QTL in each mapping population ranges from one to six, and most of these mapped loci are of large phenotypic effect, explaining 5–27% of the total trait variance. In general, QTL for germination salt tolerance and those for the vegetative stage do not overlap, indicating different mechanisms are involved in salt tolerance for these the two stages. The most common phenotype measured was the percentage of germination/cotyledon greening, although two studies (DeRose-Wilson and Gaut 2011; Joosen *et al.* 2010) scored the time to reach 50% germination (*i.e.*, germination speed). DeRose-Wilson and Gaut (2011) mapped three QTL for germination speed under salt from Col × Ler RILs, on chromosomes 3 and 5, and they only partially overlap with the percentage germination/cotyledon greening QTL. Likewise, Joosen *et al.* (2010) mapped four germination speed QTL under salt from Bay × Sha RILs, also on chromosomes 3 and 5, partially overlapping QTL they mapped for other salt-tolerant germination traits. These findings indicate that germination speed may contribute to germination salt tolerance trait.

We used the X-QTL mapping platform to examine the genetics of germination speed. Unlike previous QTL studies, this approach potentially has higher power (with a sample size of ∼100,000 F_3_ recombinants), and can provide a more comprehensive catalog of loci compared to previous mapping studies. We observed increased phenotypic variation compared to the no-salt condition (*i.e.*, a range in germination time of 15 d instead of 72 hr), and changes in the schedules of each developmental stage (testa rupture, radicle protrusion, cotyledon greening, and cotyledon expansion) during germination.

We identified eight QTL for early germination under extreme salinity in our mapping population, and four QTL for late germination. We compared the QTL maps of germination speed under saline conditions to that observed in no-salt conditions (Yuan *et al.* 2016). We identified that the QTL on the distal arm of chromosome 4 in saline conditions (SaltEarlyq4.1) overlaps with a germination speed QTL we previously identified under no-salt germination conditions (Yuan *et al.* 2016). We also found that SaltEarlyq5.2 and SaltLateq5.1 overlap with a salt germination speed QTL previously mapped by other groups (DeRose-Wilson and Gaut 2011; Joosen *et al.* 2010). This suggests that some of the early germination response to salt stress may simply be due to genes associated with a general acceleration in germination timing.

However, most peaks do not overlap between the salt and normal conditions, and we identified more QTL in salt compared to no-salt conditions (12 *vs.* five loci). These results suggest there are polymorphic genes between the Bs-2 and Col-0 accessions that remain unexpressed under no-salt conditions, but are observed in the novel saline environment. This release of cryptic genetic variation leads to increased variation in germination speed between the founder accessions.

When we attempted to map the loci underlying germination ability under salt stress (by pooling all germinants from all time cohorts), we detected few regions that exhibited an allele frequency bias, and none of them were statistically significant. This might be due to the contrasting genes/alleles at different time points in the germination process.

This appears to be the case, as we found that the genetic architecture of germination under salt stress differs in early *vs.* late germinating cohorts, suggesting multiple pathways to salt tolerance during the developmental period between testa rupture and cotyledon expansion. Moreover, QTL on chromosomes 2 and 4 exhibit opposite allele frequency biases in early and late germination cohorts. There are two possible reasons for this observation: either these are due to different genes with opposite effects in early *vs.* late germination, or time-dependent allelic effects of the same genes. Establishing the genetic mechanism for this phenomenon will have to await isolation of the causal genes.

We found that some of the QTL we identified appear to overlap with QTL identified in previously published *A. thaliana* germination salt tolerance QTL studies. Determining exact overlap is difficult. Given that positions of most of the previously identified QTL were reported on a genetic (rather than physical) map, we are only able to approximate the extent of overlap between studies. Nevertheless, it appears that the SaltEarlyQ1.1 and SaltEarlyQ5.2 overlaps with eight previously identified QTL from various studies, while SaltEarlyQ4.1 and SaltLateQ5.1 overlaps with three QTL each (see Table 2 for a full list of overlapping QTLs). However, many of other QTL we detected by X-QTL mapping do not overlap with any known QTL.

It is noteworthy to compare our approach with another bulk segregant mapping study of germination salt tolerance in *A. thaliana* (Guo *et al.* 2016). Although the approaches behind our two studies are similar, we used different founder accessions, a much larger sample size, and more stringent selection threshold. Nevertheless, of the nine QTL they reported, five colocalize with QTL on chromosomes 1, 2, and 5 identified in our study. The three QTL on chromosome 1 are in close proximity, and other classical QTL studies cannot separate these three loci; in both our and the Guo *et al.* (2016) study, however, these QTL are well-resolved, highlighting the power and resolution of high-throughput selection/bulk segregant analysis in mapping QTL.

A major goal of QTL analysis is to identify specific genes that underlie complex traits. Although functional identification of specific loci is beyond the scope of this study, we attempted to identify plausible positional candidate genes by examining the annotations of genes that fall within the QTL peaks (Table 2). We considered a gene to be a strong candidate if it had annotation attributed to salt/dehydration stress response, germination, ABA metabolic/signaling pathways, and GA metabolic/signaling pathways. The choice of relevant gene annotation was based on the osmotic and ionic stress components of extreme salinity (Munns and Tester 2008), as well as the key roles of the hormones ABA and GA in both germination (Bewley 1997) and stress response (Nakashima and Yamaguchi-Shinozaki 2013). Eighteen genes met these criteria. We then looked for tissue/stage-specific gene expression pattern for these genes using ePlant (http://bar.utoronto.ca/~dev/eplant). With these criteria, we identified 10 genes in four QTL regions that had appropriate functional annotation and had either high expression levels in mature seeds, or a dramatic change in expression level from dry to imbibed seeds (see Table 3). Future functional validation by quantitative complementation of the alleles for these genes will be necessary to conclusively demonstrate whether any of these loci indeed drives the phenotypic difference between the parental accessions.

**Table 3:** Candidate genes within germination speed under salt X-QTL regions

AGI	Gene Name	QTL	Description	Citation
AT1G72770	*HAB1*	SaltEarlyQ1.4	Protein phosphatase 2C, mutant has ABA hypersensitive inhibition of seed germination	Kuhn *et al.* 2006
AT2G29090	*CYP707A2*	SaltLateQ2.1	Protein involved in ABA catabolism, plays a major role in the rapid decrease in ABA levels during early seed imbibition	Liu *et al.* 2009
AT2G29380	*HAI3*	SaltLateQ2.1	Highly ABA-induced PP2C gene 3	Schweighofer *et al.* 2004
AT2G31660	*SAD2*	SaltLateQ2.1	Encodes an importin β-domain family protein likely to be involved in nuclear transport in ABA signaling	Verslues *et al.* 2006
AT4G28520	*CRU3*	SaltEarlyQ4.1	Encodes a 12S seed storage protein whose phosphorylation state is modulated in response to ABA in *Arabidopsis thaliana* seeds	Lin *et al.* 2013
AT4G30660	*N/A*	SaltEarlyQ4.1	Low temperature and salt responsive protein family	www.arabidopsis.org
AT5G45830	*DOG1*	SaltEarlyQ5.2	*DELAY OF GERMINATION 1*. Causal gene of a quantitative trait locus involved in the control of seed dormancy	Bentsink *et al.* 2006
AT5G51760	*AHG1*	SaltEarlyQ5.2	ABA-hypersensitive germination 1, a putative PP2C. Expressed in seeds and functions in seed development and germination	Nishimura *et al.* 2007
AT5G52300	*LTI65*	SaltEarlyQ5.2	Encodes a protein that is induced in expression in response to water deprivation such as cold, high-salt, and desiccation via ABA	Nakashima *et al.* 2006
AT5G54390	*AHL*	SaltEarlyQ5.2	Encodes a 3′-phosphoadenosine-5′-phosphate (PAP) phosphatase that is sensitive to physiological concentrations of Na^+^	Gil-Mascarell *et al.* 1999

AGI, Arabidopsis Genome Initiative.

In this study, we used the X-QTL mapping platform and a time series experimental design to map germination speed QTL under a novel abiotic environment. The mapping population size (∼100,000 F_3_ recombinants) is by far the largest used in mapping studies for multicellular organisms, which can potentially lead to higher mapping power and precision (Ehrenreich *et al.* 2010; Beavis 1997). The combination of salt stress that increased the phenotypic range of germination speed variation, the large mapping population, and the time-series sampling design enabled us to isolate three temporal germination cohorts. Unlike previous studies, the isolation of these germination cohorts allowed us to examine dynamic germination behavior under salinity, and to focus our attention on how intrinsic variation of germination speed responds to salinity, rather than simply mapping whether or not seeds germinate under salinity.

Our findings suggest that the range of phenotypic variation in germination speed increases upon saline treatment, consistent with previous studies that point to a decrease in genetic canalization under novel (stressful) environments (Waddington 1942; Waddington 1953; Paaby and Rockman 2014). We also observe intraspecific standing genetic variation that is observed only in saline conditions, indicating cryptic genetic variation that exhibits a clear G×E interaction. Our results suggest that cryptic genetic variation may play a crucial adaptive role in facilitating species range expansions, exploitation of new niches or response to climate change. Identification of the causal genes underlying this cryptic genetic variation in abiotic stress tolerance may illuminate the mechanisms that can underlie response to novel environments, and lead to greater understanding of adaptation that may accompany species dispersal. Moreover, many of these genes may also prove useful in an agricultural context, providing new genes that can be used to improve crop performance in marginal environments.

## 

## Supplementary Material

Supplemental Material
