# Supplementary material for: Cryptic Genetic Variation for Arabidopsis thaliana Seed Germination Speed in a Novel Salt Stress Environment
Source: G3 (Bethesda). 2016 Aug 18;6(10):3129–38. doi: 10.1534/g3.116.033944 (PMC5068935; doi:10.1534/g3.116.033944)
Supplement: Supplemental Material [file supp_6_10_3129__index.html]

Cryptic Genetic Variation for Arabidopsis thaliana Seed Germination Speed in a Novel Salt Stress Environment — Supplemental Material 

# Cryptic Genetic Variation for *Arabidopsis thaliana* Seed Germination Speed in a Novel Salt Stress Environment

## Supplemental Material for Yuan *et al.*, 2016

**Files in this Data Supplement:**

- File S1 - Bulk-segregant microarray genotyping data of early, medium and late salt tolerant germination cohorts, and the entire salt germinant pool. (.zip, 96 MB)
